# Trust in the Provider and Glaucoma-Related Blindness

**DOI:** 10.5402/2012/393917

**Published:** 2012-06-20

**Authors:** Kelly W. Muir, Brian Alder, Anitra Thomas, Sara S. Crowell, Sandra S. Stinnett, Paul P. Lee

**Affiliations:** ^1^Department of Ophthalmology, Duke Univeristy Medical Center, 2351 Erwin Road, P.O. Box 3802, Durham, NC 27710, USA; ^2^Durham VA Medical Center, 508 Fulton Street, Durham, NC 27705, USA; ^3^Department of Ophthalmology, Univeristy of California at Los Angeles, Los Angeles, CA 90095, USA; ^4^Department of Ophthalmology and Visual Sciences, University of Michigan, 1000 Wall Street, Ann Arbor, MI 48105, USA

## Abstract

*Purpose*. We hypothesized that lower trust in the physician is associated with worse visual outcomes in glaucoma. *Methods*. Subjects completed the Trust in Provider Scale (TPS) and performed visual field tests at least two years apart. The primary outcome was glaucoma-related blindness. *Results*. Subjects with glaucoma-related blindness scored lower on the TPS (74.9 ± 7.4, *n* = 21) than subjects without blindness (78.8 ± 6.9, *n* = 39; *P* = 0.04). In white subjects, TPS scores were similar for blind (77.1 ± 7.7, *n* = 12) versus not blind subjects (76.4 ± 6.7, *n* = 10; *P* = 0.82). For nonwhite subjects, TPS scores were lower for blind (72.0 + 6.2, *n* = 9) versus not blind subjects (79.6 ± 6.9, *n* = 29; *P* = 0.005). In multiple regression models, the interaction between race and trust was significant (*P* = 0.007), indicating that the increase in odds for blindness with each unit decrease in TPS score was different for white versus nonwhite subjects. *Discussion*. Glaucoma patients' trust in the physician is associated with glaucoma-related blindness in this study. The association between lower trust in the physician with blindness in patients of nonwhite race deserves further attention as we strive to reduce disparities in visual outcomes.

## 1. Introduction

African Americans are at least three times more like to have glaucoma than white Americans [1] and blindness from glaucoma is at least five times more common in African Americans than in white Americans with the disease [2]. Socioeconomic factors including educational attainment, income, and employment status are related to racial disparity in visual outcomes for patients with glaucoma, but do not completely explain this disparity [3]. Undertreatment of glaucoma in African Americans [4] as well as biologic differences in disease severity may contribute to disparate outcomes. Elements of the patient-provider relationship may also influence disease progression [5].

Trust is one important element of the patient-provider relationship. Aspects of trust include the provider's trust in the patient, the patient's trust in the healthcare system, and the patient's trust in the provider. The patient's trust in the provider may affect the way the patient manages his or her disease. For example, patients who report lower levels of trust in their provider are more likely to forego their glaucoma medications when under financial constraints than are patients with higher levels of trust in their provider [6]. 

We found that in a population of subjects with glaucoma treated in an academic medical center, levels of trust in the provider were generally high, but nonwhite subjects reported lower levels of trust in the provider than did white subjects [7]. Considering the disparity in trust levels between African American and white patients with glaucoma and the racial disparity in visual outcomes, we questioned if levels of trust in the provider might be associated with visual outcomes for patients with glaucoma. Accordingly, we studied the association between trust in the provider and glaucoma-related blindness in a cohort of glaucoma patients followed for at least two years. 

## 2. Methods

The original study was designed as a cross-sectional patient survey and concomitant chart review. As reported previously, [7] subjects with open-angle glaucoma and cared for by one of four glaucoma subspecialists (one white, one African American, one Chinese American, and one of Southeast Asian descent) were approached regarding study participation. Subjects were excluded if visual field tests were not present in the medical record. Subjects who provided informed consent were asked to complete survey instruments, including the Mini-Mental State Exam (MMSE) [8] and the Trust in Physician Scale (TPS). Subjects who scored less than 18 on the MMSE were excluded due to concern that diminished cognitive status would confound the results of the survey instruments. The TPS is an 11-item, self-administered questionnaire scored 1–100, with 100 indicating greatest trust. Items included in the TPS were derived from patient interviews. Test items are answered in a 5-point Likert format. Internal reliability is excellent (Cronbach alpha = 0.90) [9] and test-retest reliability has been validated [10]. All surveys were administered by the same research coordinator. Approval of the Institutional Review Board was obtained to review the medical records of study participants. 

Subjects were included in the study if two sets of visual field tests were available separated by at least two years. Glaucoma-related blindness was defined for each eye separately as visual field defects within 20 degrees of fixation on Humphrey Visual Field testing. Demographic data were collected from the medical record and included age, gender, and self-reported race.

### 2.1. Statistical Methods

Initially, descriptive statistics were obtained, (means, standard deviations, medians for continuous data, and frequencies and percentages for categorical data). Group means were compared with unpaired students' *t*-test. The relationship between trust, race, and progression to blindness was examined in a multiple regression model. All statistical analyses were performed using SAS E-Guide Version 4 for Windows (SAS Institute, Cary, NC). Two-sided *P*-values at the standard 0.05 level were used to determine statistical significance.

## 3. Results

Of the 195 subjects who participated in the original study, 60 subjects met inclusion criteria for the follow-up survey. Subjects included in the current study did not differ from subjects enrolled in the original study but excluded from the current study either by TPS score (*P* = 0.143) or by severity of glaucoma at baseline as judged by mean deviation of visual field in the worse-seeing eye (*P* = 0.410). The characteristics of the subjects are presented in Table 1. All but one of the nonwhite subjects self-identified as African American.

Scores on the TPS, scaled 0–100, for ranged from 59.09 to 100.00, mean 77.42 ± 7.28, median 75.00. Subjects with glaucoma-related blindness in one or both eyes over the course of the study scored lower on the TPS (*n* = 21, TPS score 74.89 ± 7.38) than subjects without blindness (*n* = 39, TPS score 78.79 ± 6.94; *P* = 0.04). 

**Figure 1:**
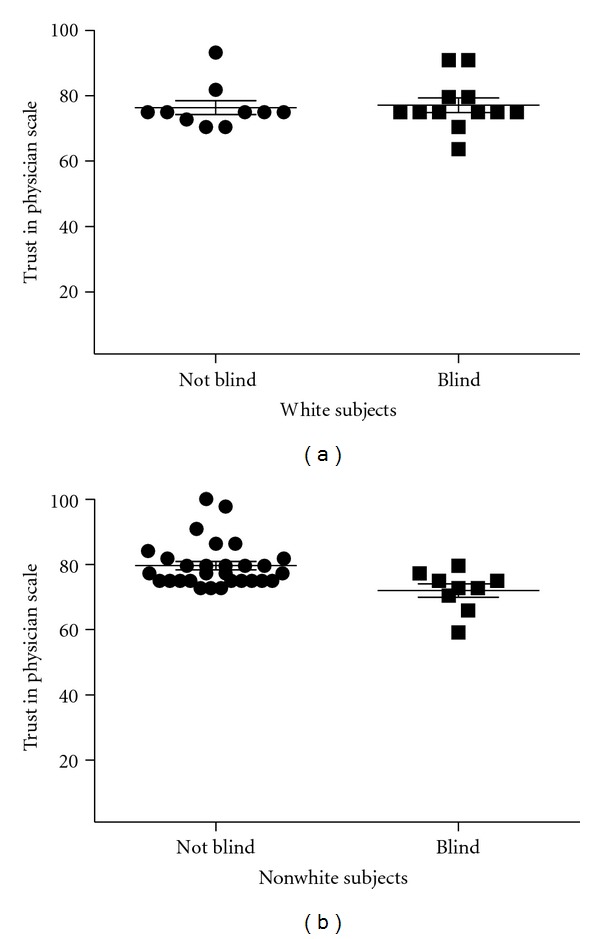
Subjects' score on the Trust in Physician Scale, an 11-item self-administered questionnaire measuring patients' trust in the provider (scaled 0–100 with 100 indicating greatest trust) is plotted on the *y*-axis. (a) For subjects self-reporting white race, the TPS scores were similar for subjects with glaucoma-related blindness in one or both eyes (*n* = 12, TPS score 77.08 ± 7.66) versus subjects without glaucoma-related blindness in either eye (*n* = 10, TPS score 76.36 ± 6.71; *P* = 0.82). (b) For subjects self-reporting nonwhite race (all but one if which identified as African American), TPS scores were significantly lower for subjects with glaucoma-related blindness in one or both eyes (*n* = 9, TPS score 72.00 ± 6.22) versus subjects without glaucoma-related blindness (*n* = 29, TPS score 79.62 ± 6.94, *P* = 0.005).

Considering white subjects only, the TPS scores were similar for blind (*n* = 12, TPS score 77.08 ± 7.66) versus not blind subjects (*n* = 10, TPS score 76.36 ± 6.71; *P* = 0.82). For nonwhite subjects, TPS scores were significantly lower for blind (*n* = 9, TPS score 72.00 ± 6.22) versus not blind subjects (*n* = 29, TPS score 79.62  ±  6.94, *P* = 0.005; Figure 1). 

In a multiple regression model including race and TPS score as explanatory variables for glaucoma-related blindness, non-white race and TPS score were associated with blindness, *P* = 0.03, and AUC = 0.81. The interaction between race and trust was significant, *P* = 0.007, indicating that the increase in odds for blindness with each unit decrease in TPS score was different for white versus nonwhite subjects.

## 4. Discussion

Previously, we reported that the level of trust in the provider reported by nonwhite subjects was lower than trust levels reported by white subjects [11]. In this study of subjects with glaucoma, we found that white patients with glaucoma were more likely to experience glaucoma-related blindness than were nonwhite subjects with glaucoma, contradictory to larger studies of race and visual outcomes in glaucoma [2]. Yet in our study, lower levels of trust in the provider and presence of glaucoma-related blindness in at least one eye were significantly associated for nonwhite subjects. That is, the odds of glaucoma-related blindness was significantly higher in nonwhite versus white subjects for each unit decrease in trust in the provider. 

A positive association between decreasing trust in the provider and glaucoma-related blindness does not indicate a causal relationship. Indeed, the connection between trust and blindness may be multidirectional. Trust may influence disease progression, particularly in chronic asymptomatic diseases requiring patient self-management. For example, patients who express greater trust in their primary care physicians also report greater adherence to the prescribed medication regimen than their less trusting peers [10]. However, we did not find a relationship between trust in the provider and glaucoma medication adherence previously [7]. Measurements of trust and medication adherence are both problematic, as trust and adherence are multidimensional concepts and our metrics are not comprehensive. 

Whether or not trust influences glaucomatous progression, disease severity may influence trust. Even under treatment, up to 9% of patients with glaucoma may become blind [12] and it is likely that progressive loss of vision while under the care of an eye care provider colors the patient-provider relationship. For example, in the in-patient setting, sicker patients reports more problems with their care [13, 14].

Lower trust in both the healthcare system and in the provider from nonwhite subjects has been described in the primary care setting [15] as well as in the care of patients with glaucoma [7]. Although trust in the provider, a dimension of interpersonal trust, and trust in the healthcare system, an element of social trust, are not synonymous, and the two concepts are difficult to tease apart. Unfortunately, there is historical precedent, such as the mistreatment of African American men in the Tuskeegee Syphilis Study contributing to a lack of trust in the healthcare system [16]. 

This study is limited by the sample size and by the nature of the academic, referral practice from which the sample was selected. The prevalence of glaucoma-related blindness was higher in this group of patients than in the general population of patients with glaucoma [12]. The measurement of trust is also problematic, even in an ideal scenario. Although the TPS demonstrates excellent psychometric properties, [10] trust may be a dynamic attribute and, in this study, was only measured at baseline. Nevertheless, despite overall high levels of trust, lower levels of trust were apparent, especially for nonwhite subjects with poor visual outcomes. 

## 5. Conclusions

Unfortunately, racial disparities in outcomes for chronic eye disease persist. African Americans are twice as likely to be bilaterally blind than white Americans [17]. As we strive to eliminate racial disparities in visual outcomes, it is important to consider the patient-provider relationship. Although variables such as trust and communication are difficult to quantify, such variables are also potentially modifiable. Trust in the provider is correlated with the patient's appraisal of the provider's communication skills, the quality of interpersonal interaction, and the provider's knowledge about the patient [18]. In terms of linking the provider-patient interaction with glaucoma management, we know that different styles of communication are associated with variable medication adherence [5]. Providers' interpersonal-communication skills can be improved through training [19, 20]. Perhaps improving the quality of the patient-provider relationship will provide a small step in our efforts to reduce racial disparities in glaucoma care. 

## Figures and Tables

**Table 1:** Characteristics of subjects.

	*N* (%)
Gender	
Male	27 (45)
Female	33 (55)
Race	
White	21 (35)
African American	38 (63)
Other	1 (2)
Blind in one or both eyes	21 (35)

	Mean + SD; median

Age at baseline	71.9 ± 9.7; 73.5
Mean deviation of visual field in the worse eye	−9.1 ± 9.0; 6.0
